# Identification of phosphorylation site on PARP1 mediating its cytosolic translocation in virus-infected HeLa cells

**DOI:** 10.1016/j.xpro.2022.101808

**Published:** 2022-11-02

**Authors:** Fei Wang, Ming Tong Ma, Junfang Xu, Haipeng Liu

**Affiliations:** 1 Shanghai Key Laboratory of Tuberculosis, Shanghai Pulmonary Hospital, Tongji University School of Medicine, Shanghai 200433, China; 2 Department of Microbiology and Immunology, Tongji University School of Medicine, Shanghai 200072, China; 3 Clinical Translation Research Center, Shanghai Pulmonary Hospital, Tongji University School of Medicine, Shanghai 200433, China; 4 Central Laboratory, Shanghai Pulmonary Hospital, School of Medicine, Tongji University School of Medicine, Shanghai 200433, China

**Keywords:** Cell biology, Cell separation/fractionation, Mass spectrometry, Microscopy, Molecular biology, Signal transduction

## Abstract

Poly (ADP-ribose) polymerase 1 (PARP1) localization is controlled by its phosphorylation state. Here, we describe a protocol to monitor PARP1 subcellular localization in HSV-1-infected HeLa cells using immunofluorescence microscopy and cytoplasmic/nuclear fractionation. We detail steps to identify phosphorylation sites on PARP1 using conserved motif analysis and mass spectrometry. This protocol can be applied to the study of other protein phosphorylation events in other cell types.

For complete details on the use and execution of this protocol, please refer to Wang et al. (2022).

## Highlights

• Evaluate PARP1 cellular distribution by confocal microscopy and cell fractionation
• Identification of PARP1 phosphorylation sites by mass spectrometry
• Validation of PARP1 phosphorylation by site-directed mutagenesis

## Abstract (editor highlights)

Publisher’s note: Undertaking any experimental protocol requires adherence to local institutional guidelines for laboratory safety and ethics.

## Graphical abstract

**Figure:**
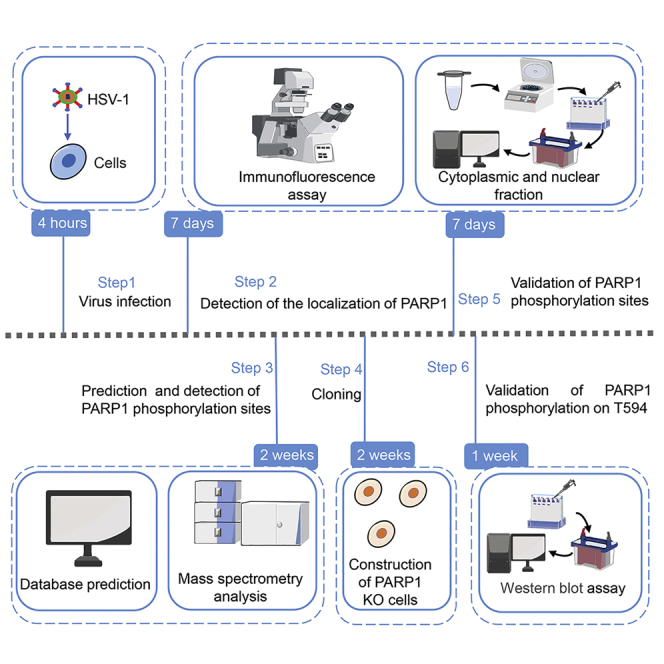

## Before you begin

### Cell culture

**Timing: 2 days**

1. Culture human cervical cancer cells (HeLa; ATCC CCL-2) and monkey kidney epithelial cells (Vero; ATCC CCL81.4) in Dulbecco’s Modified Eagle’s Medium (DMEM; Gibco) supplemented with 10% (v/v) heat-inactivated fetal bovine serum (FBS; Gibco), 1% (v/v) penicillin–streptomycin, 1 mM of sodium pyruvate, 2 mM of L-glutamine, 10 mM of HEPES buffer, and 50 μM of 2-mercaptoethanol (all from Gibco). Maintain cells at 37°C in 5% CO_2_.

***Note:*** All cells were free of mycoplasma confirmed by the LookOut Mycoplasma PCR Detection Kit (MP0035, Sigma-Aldrich).

2. Seed cells in 15 mm glass bottom cell culture dishes (NEST, Jiangsu, China) (about 3 × 10^5^ cells/dish) to be 70%–80% confluent at transfection. Dilute 2–5 μL lipofectamine 2000 Reagent and 1–2 μg corresponding PARP1 plasmids in 125 μL Opti-MEM Medium respectively. Add diluted DNA to diluted Lipofectamine 2000 Reagent (1:1 ratio) and incubate for 5 min. Add DNA-lipid complex to cells. Incubate cells at 37°C for 36 h and infected with HSV-1 virus (MOI = 1).

### Virus amplification

**Timing: 7 days**

Culture Vero cells in a 500 cm^2^ culture flask. When the cell confluency reaches 80%–90%, replace the complete DMEM medium with serum-free DMEM medium, and infect the cells with HSV-1 (MOI = 0.001). After 1 h, replace it with complete DMEM medium (without Penicillin-Streptomycin Solution). 3–4 days later, 90% of the cells are diseased and then collected in a 50 mL centrifuge tube. Freeze cells at −80°C for 1 h and thaw at 25°C for 3 times, collect the supernatant and centrifuge at 120 × *g* for 5 min. Measure the titer of HSV-1 in the supernatant through means of 50% of the tissue cultures infectious dose (TCID50) on Vero cells (Chen et al., 2013; Zhou et al., 2016).

### Construction of PARP1 KO cells

**Timing: 14 days**

Use LentiCRISPRv2 vectors to generate knockout (KO) cells (Sanjana et al., 2014). Transfect the HEK293T cells with pMD2.G, pSPAX2 and lentiCRISPRv2 harboring the guide (g)RNA that target human PARP1 (5′-CGAGTCGAGTACGCCAAGAG-3′) at a ratio of 1:3:4. Harvest the lentiviruses after 48 h post transfection, collect and store the cell culture supernatant at 4°C. Collect cell supernatant again after adding complete DMEM medium (without Penicillin-Streptomycin Solution) for 24 h. Mix the two supernatants, centrifuge at 120 × *g* for 5 min to remove cell debris, and store at −80°C. Mix the virus-containing medium with an equal volume of complete DMEM medium (without Penicillin-Streptomycin Solution) and add to HeLa cells. 48 h later, select the infected cells with puromycin (4 μg/mL) for 48 h–72 h and acquire single-cell cloning through serial dilutions in a 96-well plate. Confirm the deletion of target gene in KO cells by western blot using anti-PARP (46D11) (CST, Danvers, MA) at 1:1000 dilution. See also troubleshooting 1, 2.

***Note:*** Before selection, the optimal concentration of puromycin could be determined by culturing HeLa cells in medium with increasing concentration of puromycin at a range. During transfection, an empty lentiCRISPRv2 control should be designed.

### HA, HA-PARP1, HA-PARP1 (T594A) reconstituted cells

**Timing: 14 days**

Transfect the *PARP1*^−/−^ HeLa cells by lipofectamine 2000 (Thermo Fisher Scientific, Waltham, MA) with pcDNA3.1-HA, pcDNA3.1-HA-PARP1 or pcDNA3.1-HA-PARP1 (T594A), respectively. At 48 h following transfection, select cells with G418 (800 μg/mL) for 6–7 d. At 48–72 h post removing G418, single-cell cloning was acquired through serial dilutions in 96-well plate. Confirm the stably selected cells through western blot and use them for further experiments. See also troubleshooting 3.

***Note:*** Concentration of G418 is very important for screening positive cells. The optimal concentration of G418 should be determined.

### Preparation of phospho-PARP1^T594^ antibody

**Timing: 2 months**

Raise a rabbit polyclonal antibody against PARP1 phosphorylated at T594 (p-PARP1^T594^) in collaboration with Abclonal Biotech. Immunize 3 rabbits with peptide c (KLH)-591RVG (p-T) VIGSNK600 (in which ‘c(KLH)’ indicates keyhole limpet hemocyanin fused through cysteine, and ‘p-T’ indicates phosphorylated threonine at a ratio of 1:1. Use the non-phosphorylated peptide c (KLH)-591RVGTVIGSNK 600 for antibody purification and detection.

## Key resources table

**Table:** 

REAGENT or RESOURCE	SOURCE	IDENTIFIER
**Antibodies**		

Anti-PARP (46D11). Working dilution: 1:1000 for WB, 1:100 for IF.	Cell Signaling Technology	Cat# 9532; RRID: AB_659884
Anti-Tubulin. Working dilution: 1:1000 for WB	Cell Signaling Technology	Cat# 2148; RRID: AB_2288042
Anti-Histone H3. Working dilution: 1:1000 for WB	Cell Signaling Technology	Cat# 4499; RRID: AB_10544537
Goat Anti-Rabbit IgG Antibody, HRP conjugate. Working dilution: 1:5000 for WB	Millipore	Cat# 12-348; RRID: AB_390191
Goat Anti-Mouse IgG Antibody, HRP conjugate. Working dilution: 1:5000 for WB	Millipore	Cat# 12-349; RRID: AB_390192
Anti- phospho-PARP1(T594). Working dilution: 1:500–1:1000 for WB.	Wang et al. (2022)	N/A
Goat anti-Mouse IgG (H+L) Cross-Adsorbed Secondary Antibody, FITC. Working dilution: 1:1000 for IF.	Beyotime	Cat# A0568; RRID: AB_2893016
Goat anti-Mouse IgG (H+L) Highly Cross-Adsorbed Secondary Antibody, Cy3. Working dilution: 1:1000 for IF.	Beyotime	Cat# A0521
Goat anti-Rabbit IgG (H+L) Highly Cross-Adsorbed Secondary Antibody, FITC. Working dilution: 1:1000 for IF.	Beyotime	Cat# A0562
Goat anti-Rabbit IgG (H+L) Highly Cross-Adsorbed Secondary Antibody, Cy3. Working dilution: 1:1000 for IF.	Beyotime	Cat# A0516; RRID: AB_2893015

**Bacterial and virus strains**		

HSV-1 (Herpes simplex virus 1, UL30)	X. Cao (Zhejiang Univ)	N/A

**Chemicals, peptides, and recombinant proteins**		

DMEM/high glucose medium	HyClone	Cat# SH30809.01B
4% PFA	Sangon Biotech	Cat# E672002-0500
Tris base	Sangon Biotech	Cat# A600194-0500
Anti-FLAG M2 Affinity Gel	Sigma-Aldrich	Cat# A2220
Trizol	Invitrogen	Cat# 15596018
Amersham Protran (0.22 μm NC)	GE	Cat# 10600001
Glycine	Sangon Biotech	Cat# A502065
PBS	Gibco	Cat# 20012027
BSA	Beyotime	Cat# ST023
Protein A/G PLUS-Agarose	Santa Cruz Biotechnology	Cat# SC-2003
Triton X-100	Thermo Fisher Scientific	Cat# BP151-500
Penicillin-Streptomycin (10,000 U/mL)	Thermo Fisher Scientific	Cat# 15140122
Permeabilization Buffer (10×)	Invitrogen	Cat# 00-8333-56
Puromycin dihydrochloride	MCE	Cat# HY-B1743A
Fetal Bovine Serum (FBS)	Gibco	Cat# 10099-141
Protein marker	Thermo Fisher Scientific	Cat# 26620
Phosphatase inhibitor cocktail	Sigma-Aldrich	Cat# P5726
SDS	Solarbio	Cat# S8010
DTT	Sigma-Aldrich	Cat# D9779
Tween-20	Solarbio	Cat# T8220
EDTA	Solarbio	Cat# E8040
Protease inhibitor cocktail	Thermo Fisher Scientific	Cat# A32965
Lipofectamine 2000 Reagent	Thermo Fisher Scientific	Cat# L11668019
Lipofectamine 3000 Reagent	Thermo Fisher Scientific	Cat# L3000015

**Critical commercial assays**		

Cell Fractionation Kit	Cell Signaling Technology	Cat# 9038
LookOut Mycoplasma PCR Detection Kit	Sigma-Aldrich	Cat# MP0035

**Deposited data**		

Original western data and microscopy data for figures	This paper	https://doi.org/10.17632/vyyd45m6tr.1

**Experimental models: Cell lines**		

Vero	ATCC	Cat# CCL81.4; RRID: CVCL_0059
HEK293T	ATCC	Cat# CRL-11268; RRID: CVCL_1926
HeLa	ATCC	Cat# CCL-2; RRID: CVCL_0030

**Recombinant DNA**		

pcDNA3.1-HA-PARP1(T594A)	This paper	N/A
pcDNA3.1-HA- PARP1	This paper	N/A
pcDNA3.1-Flag- PARP1	This paper	N/A

**Software and algorithms**		

ImageJ (Fiji)	NIH	https://imagej.nih.gov/ij/; RRID: SCR_002285
Prism 8	GraphPad	https://www.graphpad.com/; RRID: SCR_002798

**Other**		

Cell culture dish (10 cm)	Corning	Cat# 430167
CellCarrier-96 plate	Corning	Cat# 3599
CellCarrier-6 plate	Corning	Cat# 3516
15 mm glass bottom cell culture dishes	NEST	Cat# 801002

## Materials and equipment

**Table:** Buffer A

Reagents	Final concentration	Stock concentration	Amount
HEPES (pH 7.9)	10 mM	0.5 M	1 mL
MgCl_2_	2 mM	2 M	50 μL
KCl	10 mM	2 M	250 μL
EDTA	100 μM	250 mM	20 μL
NP-40	0.2%	N/A	100 μL
H_2_O	N/A	N/A	48.58 mL

Stocks can be kept at −20°C for 1–3 months.

**Table:** Wash buffer

Reagents	Final concentration	Stock concentration	Amount
HEPES (pH 7.9)	10 mM	0.5 M	1 mL
MgCl_2_	2 mM	2 M	50 μL
KCl	20 mM	2 M	500 μL
EDTA	100 μM	250 mM	20 μL
H_2_O	N/A	N/A	48.43 mL

Stocks can be kept at −20°C for 1–3 months.

**Table:** Buffer B (stocks can be kept at −20°C for 1–3 months)

Reagents	Final concentration	Stock concentration	Amount
HEPES (pH 7.9)	20 mM	0.5 M	2 mL
MgCl_2_	1.5 mM	2 M	37.5 μL
NaCl	640 mM	2 M	16 mL
EDTA	200 μM	250 mM	40 μL
Glycerol	2.5%	10%	12.5 mL
H_2_O	N/A	N/A	19.42 mL

Stocks can be kept at −20°C for 1–3 months.

**Table:** 1×TBST (stocks can be kept at 25°C for 1 week)

Reagents	Amount (for a 2 L stock)
10×TBS	200 mL
Tween 20	2 mL
ddH_2_O	To 2 L

Stocks can be kept at 25°C for 1 week.

**Table:** Blocking buffer

Reagents	Amount (for a 100 mL stock)
BSA	5 g
1×TBST	To 100 mL

Stocks can be kept at 4°C for 1 week.

**Table:** 5× SDS protein loading buffer (stocks can be kept at −20°C for 3 months)

Reagents	Amount (for a 100 mL stock)
1 M Tris-HCl (pH 6.8)	6 mL
Glycerin	25 mL
1% Bromophenol blue solution	10 mL
10% SDS	20 mL
ddH_2_O	To 100 mL

Stocks can be kept at −20°C for 3 months.

## Step-by-step method details

### Virus infection

**Timing: 7 days for step 1**

1. Virus infection.

Propagate HSV-1 viruses and measure the titers of virus by TCID50 assay on Vero cells (Chen et al., 2013; Zhou et al., 2016). Store the virus at −80°C for 2–4 years. Infect HeLa cells with HSV-1 (MOI = 1) for indicated time.

### Detection of the localization of PARP1 after virus infection

**Timing: 3 days for step 2**

**Timing: 5 days for step 3**

2. Detection of PARP1 localization by immunofluorescence assay.
  a. Seed HeLa cells in 15 mm glass bottom cell culture dishes (NEST, Jiangsu, China) (about 3 × 10^5^ cells/dish). At 70%–80% confluency, infect cells with HSV-1 (MOI = 1) for 4 h.
  b. Discard the culture medium, wash cells twice with sterile 1× phosphate-buffered saline (PBS) (500 μL/dish).
  c. Fix the cells with 4% paraformaldehyde (PFA) (Beyotime, Shanghai, China) for 10–20 min at 25°C.
  d. Wash cells three times with 1×PBS, and treat cells with 1× permeabilization buffer (eBioscience, California, USA) for 5 min at 25°C.
    ***Note:*** The incubation time of permeabilization buffer should not be too long.
    ***Alternatives:*** 1× permeabilization buffer can be used for cell membrane permeabilization. Alternatively, cells can be treated with 0.1% Triton X-100 in 1×PBS for 10 min.
  e. Wash cells three times with 1×PBS.
  f. Block cells with 1% BSA for 30 min at 25°C.
  g. Dilute the PARP1 antibody in QuickBlock Primary antibody diluent for immunostaining (Beyotime, Shanghai, China) at 1:100 and add to the dish, then incubate for 12 h at 4°C.
  h. Wash cells 3 times with 1×PBS, and incubate with 1 mL of secondary antibody (goat anti-Rabbit Alex Fluor594 antibody) (Beyotime, Shanghai, China) (diluted 1:1,000 in 1% BSA) at 25°C for 1 h in the dark.
    ***Note:*** Light should be avoided during the incubation of secondary antibody to prevent fluorescence quenching.
  i. Incubate cells with 200 μL DAPI Staining Solution (Beyotime, Shanghai, China) at 25°C for 5–10 min in the dark.
    ***Note:*** DAPI incubation time should not be too long to avoid high fluorescent background.
  j. After 3 washes with 1×PBS, add 1 mL of antifade mounting medium. Collect images using a Leica TCS SP8 confocal laser microscopy system (Leica Microsystems, Buffalo Grove, IL) with 63× oil immersion.
    ***Note:*** It is best to observe dish with antifade mounting medium immediately. If not immediately, it is recommended to store the sample at 4°C in dark for no more than 1 week.
  k. Determine PARP1 localization in cells from different dishes. The photos of ten different microscopic fields were taken. The DAPI signal corresponds to the nucleus. The localization of PARP can be analyzed by ImageJ software (1.8.0.172) according to signal segmentation and further data analysis can be carried out using the GraphPad Prism 8 software. Data were representative of three independent experiments with similar results can be found in Figure 1A.
    ***Note:*** Avoid dish drying when washing with PBS or adding reagents in all the steps to prevent failure in experiment or collection of misleading information.

**Figure 1:**
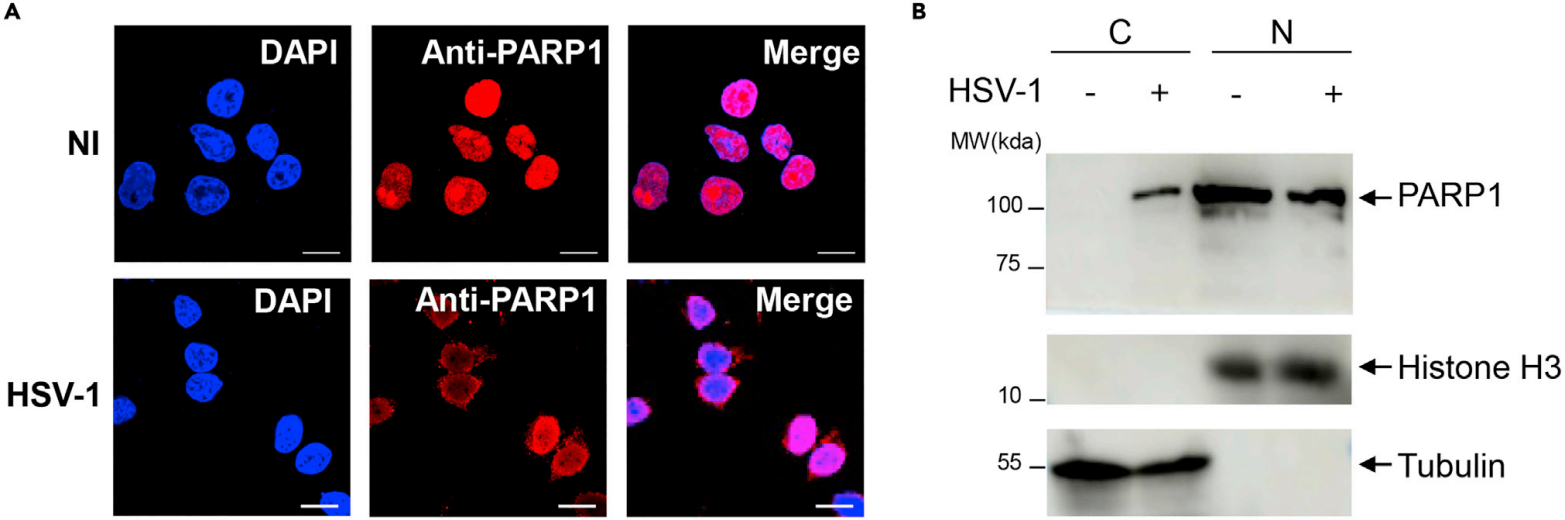
HSV-1 induced translocation of PARP1 into the cytoplasm (A) Immunofluorescence assay showing the localization of endogenous PARP1 in HeLa cells uninfected or infected with HSV-1 at MOI = 1 for 4 h. Scale bar, 10 μm. Nuclei, DAPI, blue. (B) Immunoblotting of indicated proteins in the cytoplasmic and nuclear fraction of HeLa cells uninfected or infected with HSV-1 at MOI = 1 for 4 h. Data in (A-B) are representative of n=3 independent experiments. Figures reprinted with permission from Wang et al. (Wang et al., 2022).
3. Detection of PARP1 localization by cytoplasmic and nuclear extraction.
  a. Seed HeLa cells in 6-well-plates (about 1 × 10^6^ cells/well), and culture for 24 h to reach 70%–80% confluency. Collect cells from two wells as one sample for cytoplasmic and nuclear fraction.
    ***Note:*** In our experience, to ensure sample quantity, cells from at least two wells were needed for one group.
    ***Alternatives:*** If you want to improve the sample quantity, you can choose one 10 cm dish cells as a sample for cytoplasmic and nuclear extraction.
  b. Collect cells and wash twice with 1×PBS in the 1.5 mL reaction tube;
  c. Centrifuge for 5 min at 120 × *g* and remove the supernatant completely;
  d. Add 40 μL Protease inhibitor cocktail (1 tablet in 2 mL PBS), 1 μL 1 M DTT to every 1 mL Buffer A (stocks can be kept at −20°C for 1–3 months) before use. Add 150–200 μL Buffer A supplemented with protease inhibitor cocktail and DTT to 20 μL cell pellet, place the tube on ice for 30 min, and centrifuge at 15,000 × *g* for 6 min. The supernatant is cytoplasmic extract, which is transferred to a pre-cooled 1.5 mL reaction tube.
    ***Note:*** Buffer A is best to be prepared freshly and pre-cooled before use. The nucleoplasm must be separated cleanly, and the residual liquid should be aspirated with a pipette.
  e. Wash the pellets 5 times with 500 μL wash buffer (10 mM HEPES (pH 7.9), 2 mM MgCl_2_, 20 mM KCl, 100 μM EDTA (pH 8.0)) (stocks can be kept at −20°C for 1–3 months) supplemented with protease inhibitor cocktail and DTT. Avoid pipetting the pellet, centrifuge at 1,000 × *g* for 5 min.
  f. Add Protease inhibitor cocktail 40 μL (1 tablet in 2 mL PBS) and 1 M DTT 1 μL for every 1 mL Buffer B (stocks can be kept at −20°C for 1–3 months) before use, add 50–80 μL Buffer B to the pellet, vortex the tube at high speed for 10–15 s every 10 min for a total of 30 min, and place it on ice during the period. Centrifuge the mixture at 15,000 × *g* for 15–30 min and collect the supernatant as the nuclear extract.
    ***Note:*** Buffer B is best prepared freshly and pre-cooled before adding.
  g. Denature both the cytoplasmic and nuclear fractions in 1× SDS protein loading buffer (stocks can be kept at −20°C for 3 months) at 95°C for 8 min for the following western blot analysis.
    ***Note:*** Collected samples should be stored at −20°C, if they are not tested immediately.
  h. Configure and run 10% SDS-PAGE regular gels (https://www.bio-rad.com/) (stocks can be kept at 4°C for 3 days). Electrophoresis was performed in 1× running buffer (https://www.bio-rad.com/) (stocks can be kept at 25°C for 1 week). Condensing gel electrophoresis conditions: 80 V, 30 min; separating gel electrophoresis conditions: 100 V, until bromophenol blue runs out of the separating gel;
  i. Membrane transfer: wet transfer. Transfer the protein to a 0.22 μm pore size nitrocellulose (NC) membrane in 1× transfer buffer (https://www.bio-rad.com/) (stocks can be kept at 25°C for 1 week) at 100 V for 1.5 h.
  j. Blocking: 5% skim milk (in 1×TBST) or 5% BSA (in 1×TBST) for 1 h at 25°C.
  k. Dilute the control Primary antibody (anti-IgG Rabbit antibody), Primary antibody (anti-PARP1 Rabbit antibody, anti-tubulin Rabbit antibody and Histone H3 Rabbit antibody) in Blocking buffer (stocks can be kept at 4°C for 1 day) at 1:1,000 dilution. Incubate the NC membrane with the antibody for 12 h at 4°C on a rotator.
  l. Recover the primary antibody and wash 3 times with 1×TBST (stocks can be kept at 25°C for 1 week) on a shaker; 10 min each time.
  m. Dilute the secondary antibody (Goat Anti-Rabbit IgG Antibody, HRP conjugate) in 1×TBST at 1:5,000 and incubate the membrane for 1 h at 25°C followed by washing three times with 1×TBST.
  n. Expose the gel to Supersignal West Pico developer, and then collect the image data by Amersham Imager 600 (General Electric Company, GE). The corresponding protein was detected as marker proteins to indicate the cytoplasmic and nuclear fractions, respectively (Figure 1B). See also troubleshooting 4, 5, 6.

### Validation of the phosphorylation site(s) responsible for PARP1 cytosolic translocation

**Timing: 1 h for step 4**

**Timing: 2 weeks for step 5**

4. Prediction of PARP1 phosphorylation sites.

The human PARP1 protein sequence can be downloaded from the database UniProtKB (http://www.uniprot.org/): P09874. The sites recognized *in vitro* by DNA-PK are reported to be D/E/Q-S/T-Q, with a glutamine residue on the C-terminal side being important (Lees-Miller and Anderson, 1991; Watanabe et al., 1994). We predict T325 residue of PARP1 as a potential phosphorylation site according to the conserved substrate motif of DNA-PK.

**Figure 2:**
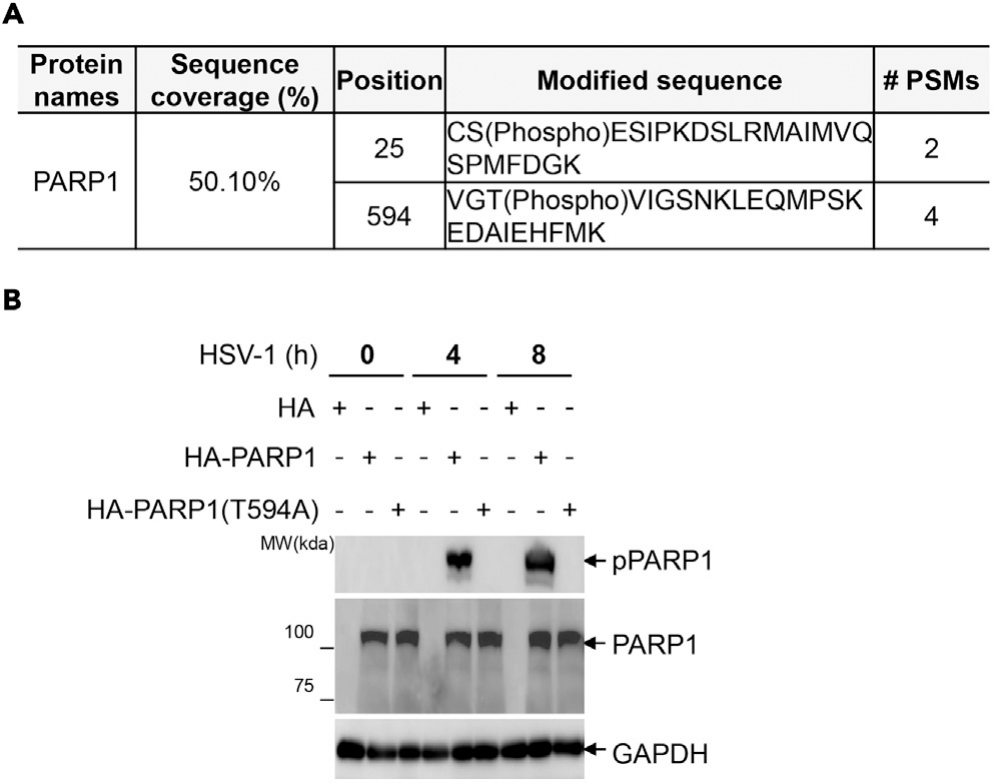
Identification and validation of phosphorylation site on PARP1 (A) T325 residue of PARP1 is predicted as a potential phosphorylation site according to the conserved substrate motif of DNA-PK. Mass spectrometry analysis of the phosphorylation sites of overexpressed Flag-PARP1 in HeLa cell infected with HSV-1 at MOI = 1 for 4 h. T594 is one of the amino acids identified. (B) Immunoblotting of indicated proteins in the lysates of PARP1 KO HeLa cells complemented with pcDNA3.1-HA, HA-PARP1 or HA-PARP1T594A and infected with or without HSV-1 at MOI = 1 for 4 h and 8 h, respectively. Data are representative of n=3 independent experiments. Figures reprinted with permission from Wang et al. (Wang et al., 2022).

5. Mass spectrometry detection of PARP1 phosphorylation sites.
  a. Seed HeLa cells in 10 cm cell culture dish (about 1 × 10^7^ cells/well) and culture for 24 h to reach 70%–80% confluency and infect with HSV-1 (MOI = 1) for 4 h.
  b. Wash cells twice with 1×PBS and lyse using 200 μL RIPA Lysis Buffer (Beyotime Biotechnology, China) supplemented with protease inhibitor cocktail (P8340, Sigma-Aldrich), 1 mM of PMSF and phosphatase inhibitor cocktail (P5726, Sigma-Aldrich). Centrifuge the lysate with table-top centrifuge at 15,000 × *g* for 10 min and the cellular debris was discarded.
    ***Note:*** After the cells were mixed with lysis buffer, they were immediately placed on a shaker at 4°C for 30 min to thoroughly lyse the cells.
  c. Wash Protein A/G agarose twice with 1 mL 1×PBS and prepared for a 50% protein A/G agarose working solution (in 1×PBS). Incubate the cell lysates with protein A/G agarose (50 μL) and anti-PARP1 antibody (5 μL) at 4°C for 12 h.
  d. Wash the beads with 1 mL 1× PBST (0.5% triton X-100 in 1×PBS) five times, discard the supernatant completely.
    ***Note:*** 1×PBST should be pre-cooled before use.
  e. Add 30–50 μL 1× protein loading buffer to the beads, 95°C, 10 min, and subject the boiled protein sample to 10% SDS-PAGE gel electrophoresis.
  f. Wash the gel (the purpose of this step is to wash off interfering substances such as SDS in the gel, reduce the staining background, and improve the staining sensitivity).
    i. Put the gel into a sterile 10 cm dish.
    ii. Add 50 mL of deionized water.
    iii. Heat it in the microwave for 3 min on high heat.
    iv. Shake for 5 min on a side-swing shaker or a horizontal shaker.
  g. Stain the gel.
    i. Carefully pour off the liquid, remove the residual liquid.
    ii. Add an appropriate amount of Coomassie brilliant blue fast staining solution (about 20 mL for each gel with a size of about 8 cm) to ensure that the staining solution can cover the gel and the liquid level is at least three gels thick.
    iii. Stain the gel on a side or horizontal shaker at 25°C. The staining time at 25°C should be 10–30 min.
    iv. Discard the staining solution until clear target protein bands appear.
    v. Add an appropriate amount of deionized water, wash off the residual staining solution, stop the staining reaction.
    vi. Take pictures and record the results.
  h. Elute the gel (This step can be performed if a background-free picture of the gel is desired).
    i. Add about 100 mL of deionized water, shake the gel on a shaker to decolorize.
    ii. Every 5–15 min, carefully pour off the liquid, add 100 mL of deionized water, and continue to decolorize on the shaker. Usually, de-staining for 30–120 min can highly reduce the background staining. See also troubleshooting 7, 8.
  i. After Coomassie brilliant blue staining and elution, the PARP1 band was cut, decolorized and subjected to mass spectrometry (MS).
    ***Note:*** The area of the cleaved band should be less than or equal to 1 cm × 1 cm (the maximum area does not exceed 1.5 cm × 1.5 cm), the target band should be clear, clearly visible to naked eyes, and there are no excess bands in the remaining molecular weight range; the cleaved band should be located in separating gel rather than concentrating gel; store in a 1.5 mL tube and keep the isolated gel moist by adding a small amount of deionized water.
    ***Note:*** If the gel concentration is relatively low and the gel is prone to be damaged due to heating, you can put the gel after electrophoresis into a container of appropriate size, add 100 mL of deionized water so that the gel is completely covered with water. Shake and wash the gel on a rotator for a total of 5 min. Washing 3–5 times can also achieve the effect of removing contaminants such as SDS in the gel. If you want to speed up the washing, you can add 100 mL of boiled deionized water, shake it on a shaker for 5 min, and wash 2–3 times in total. The purpose of microwave heating or washing with heated deionized water is to speed up the washing process and shorten the washing time. If the time is sufficient, it can be washed at 25°C.
  j. Using Proteome Discoverer 1.4 (Thermo Fisher Scientific) to obtain the resulting MS/MS data.
    i. Load the sample onto an HPLC chromatography system named Thermo Fisher Easy-nLC 1000 equipped with a C18 column (1.8 mm, 0.15 × 100 mm).
    ii. Solvent A contained 0.1% formic acid and solvent B contained 100% acetonitrile. The elution gradient was from 4% to 18% in 182 min, 18%–90% in 13 min solvent B at a flow rate of 300 nL/min.
    iii. Mass spectrometry analysis were carried out at the PTMBio Co., Ltd. (Shanghai, China) in the positive-ion mode with an automated data-dependent MS/MS analysis with full scans (350–1,600 m/z) acquired using FTMS at a mass resolution of 30,000 and the ten most intense precursor ions were selected for MS/MS.
    iv. Use higher-energy collision dissociation to acquire the MS/MS at 35% collision energy at a mass resolution of 15,000.
    v. Tandem mass spectra were searched against human PARP1 protein sequence. Trypsin/P was specified as cleavage enzyme allowing up to 2 missing cleavages.
    vi. Mass error was set to 20 ppm for precursor ions and 0.05 Da for fragment ions.
    vii. Carbamidomethyl on Cys were specified as fixed modification, oxidation on Met and phosphorylation on Ser, Thr, Tyr were specified as variable modifications. Peptide confidence was set at high, and peptide ion score was set > 20 (Figure 2A). See also troubleshooting 9, 10.

### Validation of PARP1 phosphorylation on T594

**Timing: 1 month for step 6**

6. Determine T594 as the phosphorylation site of PARP1.
  a. Reconstitute PARP1 KO HeLa cells with HA, HA-PARP1, or HA-PARP1(T594A) plasmid, respectively.
  b. Seed HeLa cells in a 6-well-plate (about 1 × 10^6^ cells/well). Culture for 24 h to 70%–80% confluency and infect with HSV-1(MOI = 1) for 4 h, 8 h.
  c. Wash cells twice with 1×PBS and denature in 1× SDS protein loading buffer at 95°C for 8 min and then were resolved by electrophoresis through a 10% SDS-polyacrylamide gel.
  d. Transfer separated proteins onto polyvinylidene difluoride (PVDF) membranes and were incubate with the anti-phospho-PARP1 T594 antibody. An enhanced chemiluminescence reagent (Thermo Fisher Scientific) was applied for immunoblotting (Figure 2B).

***Note:*** The anti-phospho-PARP1 T594 antibody should be diluted (1:100–1:1,000) to determine the optimal working concentration. The final optimal dilution of anti-phospho-PARP1 T594 antibody used for the assay was 1:500.

## Expected outcomes

Detect and quantify the localization of PARP1 and corresponding PARP1 mutants with immunofluorescence microscopy and cytoplasmic/nuclear fraction. The results were expected to demonstrate that PARP1 is able to translocate from the nucleus to the cytoplasm in response to HSV-1 infection. Based on the results from mass spectrometry analysis, we generate PARP1 phospho-dead mutants HA-PARP1(T594A) plasmid and reconstitute PARP1 KO HeLa cells with HA-PARP1, or HA-PARP1(T594A) plasmids, respectively. We then analyze the localization of PARP1 WT vs PARP1(T594A). The results were expected to determine T594 as the phosphorylation site of PARP1 responsible for its cytosolic translocation.

## Limitations

In this protocol, only HeLa cells was employed to quantitatively analyze the cytoplasm/nucleus of PARP1. Meanwhile, confocal microscopy and cytoplasmic/nuclear extraction can only reflect a rough distribution of PARP1, and a more accurate subcellular localization requires more precise experimental methods to detect. In order to better study the function to cytosolic PARP1, fusion of PARP1 with nuclear export sequence (NES), LEKLKL sequence (Fu et al., 2013; Huang et al., 2021; Kosugi et al., 2014; Sun et al., 2021) would be helpful to confine PARP1 in the cytosol (Wang et al., 2022).

## Troubleshooting

### Problem 1

In the experimental process of constructing PARP1 KO cells, no positive cells could be obtained.

### Potential solution

gRNA might not be efficient. It is recommended to check whether gRNA design has a problem and design several pairs of gRNAs.

### Problem 2

In the experimental process of constructing knockout cells, the cell state is poor.

### Potential solution

During selecting positive knockout cells with puromycin or G418, the dead cells should be removed in time, and the fresh medium or new cell culture dish should be replaced.

### Problem 3

The knockout cells or reconstituted HeLa cells grow slowly.

### Potential solution

Increase the amount of FBS in the medium. The concentration of FBS can be increased to 20%.

### Problem 4

The unspecific background signals are too high in obtaining image of immunoblotting at step 3.

### Potential solution

Proper extension of blocking time; wash the nitrocellulose filter membrane thoroughly; use appropriate concentration of primary and secondary antibody; the incubation time of the antibody should not be more than 24 h.

### Problem 5

Incomplete separation in step 3 of nucleus and cytoplasm.

### Potential solution

The cytoplasm contains nuclear components. When lysing cells, try to pipette the precipitated cells evenly. At the same time, the cleavage time is needed to be optimized, too short cleavage time would lead to incomplete cleavage, and too long cleavage time would lead to increased content of nucleus in cytoplasm.

### Problem 6

No obvious PARP1 band in step 3 of nucleus or cytoplasm fractionation.

### Potential solution

On the basis of the initial cell amount of 1 × 10^7^, increase the number of cells. Increase the anti-PARP1 antibody concentration and incubation time.

### Problem 7

Unstained band in the process of coomassie brilliant blue staining and elution at step 5.

### Potential solution

Increase the amount of loaded sample. It is recommended to load 30–50 μg of BSA as a positive control during electrophoresis.

### Problem 8

The sensitivity of the stained band is not ideal in the process of coomassie brilliant blue staining and elution at step 5.

### Potential solution

Coomassie brilliant blue fast staining solution can be used for the second staining to improve the staining effect. Prolonging the staining time can improve the detection sensitivity, and fully de-staining with deionized water can also reduce the staining background and significantly improve the staining sensitivity.

### Problem 9

There is only solvent peak in the spectrum at step 5.

### Potential solution

The injection needle is damaged; The carrier gas flow rate is too low; The sample concentration is too low; The sample is adsorbed by the column or injector bushing.

### Problem 10

Mass deviation of mass spectra at step 5.

### Potential solution

If the mass number of the mass spectrometer is off, it is time to calibrate your instrument.

## Resource availability

### Lead contact

Further information and requests for reagents may be directed to, and will be fulfilled by the lead contact, Prof. Haipeng Liu (haipengliu@tongji.edu.cn).

### Materials availability

Requests for the plasmids and cell lines generated in this study should be directed to the lead contact with a completed Materials Transfer Agreement.

## Data Availability

This study did not generate/analyze any code.
